# Control of Vocal and Respiratory Patterns in Birdsong: Dissection of Forebrain and Brainstem Mechanisms Using Temperature

**DOI:** 10.1371/journal.pone.0025461

**Published:** 2011-09-28

**Authors:** Aaron S. Andalman, Jakob N. Foerster, Michale S. Fee

**Affiliations:** McGovern Institute for Brain Research, Department of Brain and Cognitive Science, Massachusetts Institute of Technology, Cambridge, Massachusetts, United States of America; Max Planck Institute for Human Cognitive and Brain Sciences, Germany

## Abstract

Learned motor behaviors require descending forebrain control to be coordinated with midbrain and brainstem motor systems. In songbirds, such as the zebra finch, regular breathing is controlled by brainstem centers, but when the adult songbird begins to sing, its breathing becomes tightly coordinated with forebrain-controlled vocalizations. The periods of silence (gaps) between song syllables are typically filled with brief breaths, allowing the bird to sing uninterrupted for many seconds. While substantial progress has been made in identifying the brain areas and pathways involved in vocal and respiratory control, it is not understood how respiratory and vocal control is coordinated by forebrain motor circuits. Here we combine a recently developed technique for localized brain cooling, together with recordings of thoracic air sac pressure, to examine the role of cortical premotor nucleus HVC (proper name) in respiratory-vocal coordination. We found that HVC cooling, in addition to slowing all song timescales as previously reported, also increased the duration of expiratory pulses (EPs) and inspiratory pulses (IPs). Expiratory pulses, like song syllables, were stretched uniformly by HVC cooling, but most inspiratory pulses exhibited non-uniform stretch of pressure waveform such that the majority of stretch occurred late in the IP. Indeed, some IPs appeared to change duration by the earlier or later truncation of an underlying inspiratory event. These findings are consistent with the idea that during singing the temporal structure of EPs is under the direct control of forebrain circuits, whereas that of IPs can be strongly influenced by circuits downstream of HVC, likely in the brainstem. An analysis of the temporal jitter of respiratory and vocal structure suggests that IPs may be initiated by HVC at the end of each syllable and terminated by HVC immediately before the onset of the next syllable.

## Introduction

Forebrain circuits that underlie complex learned behaviors evolved after the brainstem and spinal cord circuits that generate innate behaviors such as locomotion, eye movements, and breathing [1]. How do forebrain circuits interact with this substrate of brainstem and spinal pattern generators? For example, the forebrain could act by selecting and initiating innate motor programs that are executed in brainstem and spinal circuits [2], or, alternatively, could completely override subcortical programs to fully specify the sequence of motor gestures of a learned behavior [3], [4]. Another possibility is that the control of a behavior could pass back and forth between forebrain and brainstem circuits.

Learned vocalizations are an example of a behavior that requires the integration of complex forebrain-generated elements with a behavior normally generated by the brainstem, namely respiration. In humans and songbirds, successful vocalization requires the precise coordination of respiratory and vocal motor systems [5], [6]. Much progress has been made understanding both the neural mechanisms underlying the temporal structure of songbird vocalization [7]–[11] and the neural circuits involved in respiratory control [12]–[16], but it is not yet known how these two systems are integrated during singing. In adult songbirds of many species, respiration during singing consists of a stereotyped sequence of expiratory and inspiratory pressure pulses [6], [17]–[19]. Each expiratory pulse (EP) typically contains a single song syllable, and there is a precise one-to-one relation between the pressure waveform of EPs and the syllables they contain [20]–[23]. Inspiratory pulses (IPs) in the gaps between syllables are highly reliable, and are actively driven by inspiratory muscles [16]. This rapid stereotyped alternation between EPs and IPs allows the bird to sing many seconds of uninterrupted highly stereotyped song.

**Figure 1:**
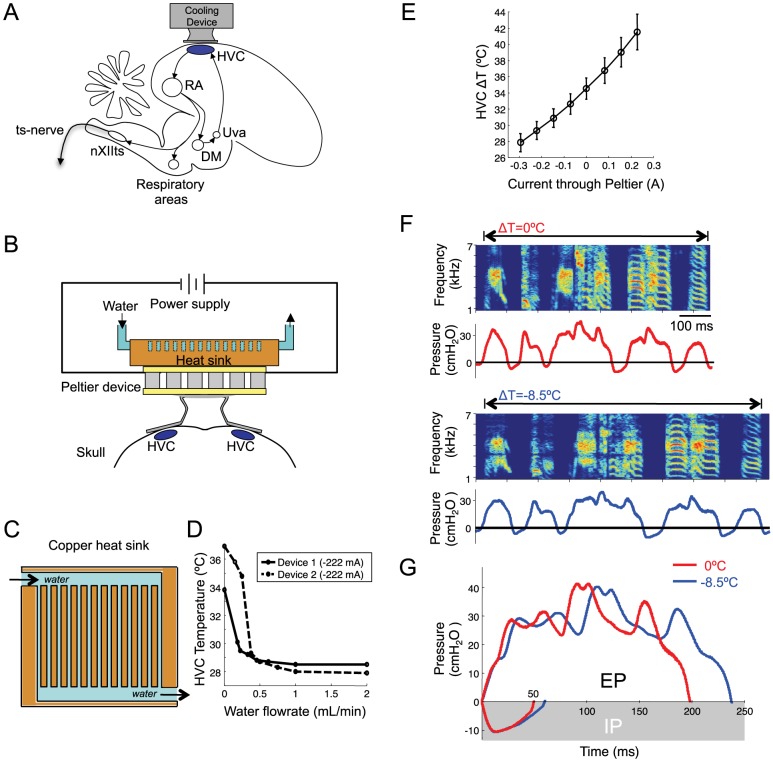
Respiratory patterns are slowed by HVC cooling. A) Sagittal schematic of the song motor pathway showing the Peltier device used for localized cooling of HVC. DM, dorsomedial nucleus of the intercollicular complex; nXIIts, tracheosyringeal part of the hypoglossal nucleus; RA, robust nucleus of the arcopallium; ts-nerve, tracheosyringeal nerve; Uva, nucleus Uvaeformis. B) Frontal-view schematic of the cooling device placed bilaterally over HVC. Water flows through a copper heat sink to efficiently dissipate heat pumped from the brain by a Peltier device. Grey blocks are alternating n- and p-doped semiconductor elements. C) Illustration of interior design of the heat sink. D) Calibration of the heat sink as a function of water flow rate was measured for the two constructed devices. A constant current was used to drive the Peltier device, and the temperature in HVC was measured at various rates of water flow through the heat sink. E) Average temperature changes in HVC as a function of current through the Peltier device (n = 6 calibration birds and 2 devices; error bars indicate standard deviation). F) Song spectrogram and thoracic air sac pressure (Bird 4) at normal HVC temperature (top) and with HVC cooled by 8.5°C (bottom). G) A single trace of an expiratory pressure pulse (EP, above) from syllable ‘c’ and an inspiratory pressure pulse (IP, below) from gap ‘c–d’, at normal and cooled HVC temperature (red and blue traces, respectively).

The remarkably precise coordination of vocal and respiratory control has led to the suggestion that the forebrain premotor circuitry controlling the temporal structure of song also controls respiration during singing [15], [16], [23], [24]. Song timing is largely controlled by forebrain nucleus HVC (used as a proper name, formerly High Vocal Center) [9]–[11], [25]. Nucleus HVC projects to another forebrain nucleus, RA (robust nucleus of the arcopallium) [26], [27], which in turn projects to respiratory areas [13]–[15], [28] and brainstem nuclei that innervate the syrinx [24], [29] (Figure 1A). Neurons in HVC that project to RA (HVC_(RA)_ neurons) generate a sparse, stereotyped sequence of bursts throughout the motif [25], and may be organized into synaptically-connected chains [30]–[32]. It has also been shown that nucleus HVC plays a critical role in the timing of song at all temporal scales. Localized temperature changes in HVC, but not in RA, result in a slowing of all components of the song by about 3% per degree Celsius of cooling [9], [33], including the fine acoustic structure within syllables, the duration of syllables and gaps, and the intervals between syllable onsets. One possibility consistent with these findings is that the sequential activation of HVC_(RA)_ neurons drives, at each moment in time, a specific pattern of activity in the downstream motor pathway resulting in the precise configuration of the vocal organ required to generate the vocal output at that moment [8], [34].

Does HVC also exert direct moment-to-moment control over the timing of respiratory events (EPs and IPs) during singing? While this is possible, it has recently been suggested that there may be a separate timing mechanism that controls the duration of IPs [35]. For example, it is possible that respiratory events are initiated by HVC, but generated elsewhere, perhaps in brainstem or midbrain respiratory centers [36], [37]. To address this question, we recorded thoracic air sac pressure in singing adult birds while bilaterally manipulating temperature in HVC to determine the effect of HVC cooling on the timing of both respiratory and vocal structure. We found that HVC cooling increased the duration of both EPs and IPs. However, whereas EPs stretched uniformly, many IPs appeared to change duration by the earlier or later termination of the underlying inspiratory event, suggesting that HVC directly controls the time course of EPs on a moment-to-moment basis, but that the time course of IPs can be strongly influenced by downstream areas. We interpret these findings in terms of the idea that HVC has a modular organization in which each syllable is related to a discrete chain of synaptically connected neurons [9]. An analysis of timing variability within gaps suggests that IPs may be initiated at the end of one HVC chain and terminated by another HVC chain at the onset of the next syllable.

## Methods

### Finches

Subjects were 10 adult male zebra finches, >120 days post hatch (dph). Birds were obtained from the MIT zebra finch breeding facility (Cambridge, Massachusetts). The care and experimental manipulation of the animals were carried out in accordance with guidelines of the NIH and were reviewed and approved by the MIT Committee on Animal Care (Protocol # 0709-075-12).

### Localized cooling of HVC in singing birds

We used a custom built miniaturized solid-state heat pump (based on the Peltier effect) to provide local bilateral cooling of HVC (Figure 1B). One face of the Peltier device (Custom Thermoelectric, Part #01101-9G30-20CN) was soldered to two gold-plated silver cooling pads (each 1 mm×2 mm in area), shaped to fit over each HVC. Birds were anesthetized with 1.5–2% isofluorane in oxygen and placed in a stereotaxic apparatus (MyNeuroLabs, Inc.) with a head angle of 65 degrees (anterior skull relative to horizontal). HVC was localized stereotaxically relative to the bifurcation of the sagittal sinus (0.0 mm anterior, 2.4 mm lateral). The outer layer of skull was removed over HVC in an area slightly larger than the size of the cooling pads. The inner layer of skull was thinned before cooling pads were gently pressed against the inner layer of skull. To increase thermal contact between the cooling pad and the skull, a thin layer of uncured Kwik-Kast (WPI, Inc) was placed on the skull, and the cooling pads were lowered into place before the Kwik-Kast hardened.

The design and construction of the cooling device were similar to those previously described [9], [38] and used a water-cooled heat sink to efficiently remove waste heat (Figure 1C and D). Current through the Peltier device was stepped between positive (warming) current to hold HVC at normal brain temperature (40°C), and various levels of negative (cooling) current. After each current step, the temperature was allowed to stabilize for three minutes before placing a female zebra finch in front of the experimental bird to elicit directed song. Some birds (n = 3) also sang a sufficient number of bouts of undirected song to allow the effect of HVC temperature changes on directed and undirected song to be compared.

### Calibration of HVC cooling device

Two devices were built for HVC cooling. Each device was calibrated by implanting the cooling device over HVC and recording brain temperature in HVC at different distances below the cooling plate (0.5 mm and 0.75 mm). Temperature was measured by placing a miniature thermocouple (Omega, 5SRTC-TT-K-40-36) in the brain directly under the cooling plate in the right hemisphere (device 1: n = 4 birds, device 2: n = 2 birds). This was done immediately after placement of the cooling plate during the same surgery. The birds were allowed to recover from anesthesia and were then held, awake, in a soft foam restraint during the temperature measurements. Because the cooling device acts as a heat sink, warming current was required to maintain the brain at normal body temperature. Current was then stepped from this control condition to warmer and cooler current conditions. A temperature reading was taken at each current level following a three-minute period for temperature stabilization. This procedure yielded a plot of brain temperature in HVC as a function of device current (Figure 1E). We estimated that the center of HVC was approximately 0.75 mm below the cooling plate; therefore temperature measurements at this depth were used to translate electrical current into estimated HVC temperature. Note that temperature changes in HVC during surface cooling are non-uniform, with an exponential temperature increase from the cooled surface to the warmer underlying tissue (length constant ∼1.2 mm, [9]). Therefore, the calibration of temperature in HVC should be viewed as approximate.

### Calibration of water-cooled heat sink

The heat sink design was optimized given material and machining constraints [39], and was produced by machining narrow channels into a thin copper block (Figure 1C and Figure S1). The effectiveness of the Peltier cooling device as a function of water flow rate through the heat sink was calibrated (as described above) by measuring brain temperature in the brain 0.75 mm below the cooling plate as a function of flow rate. Adequate heat removal was achieved with a water flow-rate of only 1.0 mL/min (Figure 1D), significantly lower than the 15 mL/min required for the heat sink used in Long and Fee (2008).

### Recordings of thoracic air sac pressure

Thoracic air sac pressure was measured during singing using an approach based on previously described techniques [40]. A thin, flexible cannula of non-bioreactive silastic tubing (RenaSil, Braintree Scientific, Braintree, MA; 0.9 mm o.d.) was inserted through the abdominal wall into the posterior thoracic air sac on one side of the body. This tube was passed between the most posterior two ribs (1.5-mm intercostal spacing) and was sutured to one rib. Before surgery, the tip of this tubing to be placed in the air sac was secured inside of a short length (5 mm, attached with a nonbioreactive polymer, Kwik-Kast, WPI) of a larger diameter tube (2-mm OD) that was fully inserted into the air sac and helped maintain a clear internal opening of the tubing. The other end of the 0.9-mm tubing was fed to a miniature piezoresistive pressure transducer (Fujikura FPM-02PG, Japan). The transducer was held in place by a loop of thin silicone tube under the skin on the bird's back. The pressure signal was amplified directly on the device and transmitted to the recording computer through a commutator (Crist Instruments 4-TBC-25-LT; Instech 375/D/20), allowing free movement of the bird inside the cage. Before implantation, the pressure sensor output voltage was calibrated in units of cmH_2_O using a water column.

### Tracheosyringeal nerve transection

Birds were anesthetized with 2% isoflurane and the trachea was exposed at the point where it exited the clavicle. A 2 mm section of tracheosyringeal nerve (ts-nerve) was removed on each side. This procedure is similar to that previously described [41].

#### Data acquisition and analysis

A direct measurement of current through the Peltier device was recorded continuously, along with the microphone signal and air sac pressure signal (40 kHz sampling rate). Data were recorded on a PC using custom Matlab data acquisition software. Analysis was carried out using custom Matlab code.

### Syllable segmentation

Syllables were segmented based on threshold crossing of acoustic power and identified by clustering based on acoustic features. Song syllables were labeled (‘a’, ‘b’, ‘c’), individual gaps were identified by the surrounding syllables (e.g. ‘a–b’, ‘b–c’, etc.; Figure S2 and S3), and motifs were identified as the most probable sequence of syllables. Syllable renditions with contaminating noises or female calls were eliminated from the analysis.

Precise syllable onset and offset times were determined from the sound amplitude as follows: The audio signal was preprocessed with a 1–4 kHz band pass filter (−50 dB suppression at 700 Hz from band edges). The sound amplitude was computed by squaring the audio signal, low-pass filtering below 30 Hz (−40 dB suppression at 700 Hz), and taking the logarithm. The second derivative was calculated numerically using a time step of 0.025 ms and smoothed with a box filter of 2.5 ms width. A 1 ms region around the syllable onset/offset time was determined from a threshold crossing of the sound amplitude, and the precise onset and offset times were taken from the peak of the second derivative within this region. Only renditions that displayed an identifiable peak in the signal were used for this part of the analysis. All syllable onsets and offsets were verified by eye for accuracy.

### Identification of IPs and EPs

An IP is a period of negative thoracic air sac pressure, as determined by the signal from the pressure sensor. Because this voltage signal demonstrated a small, slow drift over the timescale of minutes, perhaps due to ambient temperature changes, the voltage value associated with zero pressure was estimated for each song file using the histogram of pressure values. Because eupneic breathing occurred before and after each song bout (Figure S4), this histogram had clear peaks associated with the inspiratory and expiratory phases of breathing. The zero point was chosen as the midpoint between these peaks. EP durations were calculated as the time between the positive-going zero crossing of the pressure and the subsequent negative-going zero crossing. IP durations were defined symmetrically as the time between the negative-going zero crossing and the subsequent positive-going zero crossing of pressure.

### Fractional stretch of song motifs

A calculation of HVC temperature-dependent motif stretch was done for each bird. The duration of each rendition of the song motif was calculated, as was the average motif duration in the control temperature condition (normal brain temperature). For the calculation of fractional motif stretch, the duration of each motif rendition (from all temperature conditions) was divided by the average control motif duration. The resulting normalized durations, as a function of temperature, were fit to a line. The slope of the best fit line (multiplied by 100) gave the fractional change in motif duration per degree temperature change in units of %/°C (‘motif stretch’). For Birds #1,3,4,7 and 8, the motif stretch was −3.32, −3.05, −3.47, −2.17, and −1.92%/°C, respectively – yielding an average stretch of −2.79±0.31%/°C, not significantly different from the previously reported value of motif stretch (−2.83±0.22%/°C, Long and Fee, 2008). In this paper, we are interested in quantifying the relative stretch of different components of the song, rather than the overall motif stretch, which has been published earlier. Thus, unless otherwise specified, all reported temperature-dependent stretch values are normalized by the motif stretch of the same bird.

We suspect that the bird-to-bird variation in motif stretch as a function of HVC temperature was largely due to variations in the effectiveness of the cooling device across different birds (perhaps due to variations in the thickness of the thinned skull below the cooling plates), rather than underlying differences in the response of the HVC circuit. Thus, we took the additional step of using the measured motif stretch as a correction to the relation between temperature and device current estimated from the calibration experiments. For example, for Bird 8, which had a motif stretch of −1.92%/°C, we suspect that the temperature changes achieved in HVC were less than in the calibration bird for the reasons outlined above. We therefore reduced our estimated temperature changes for this bird by a factor of 67.8%, given by the ratio of the observed HVC stretch (−1.92%/°C) and the average of all HVC stretch measurements (−2.81%/°C) made from birds in this study and a previous study [9]. A similar correction was made separately for each bird. Because stretch values were normalized by motif stretch (see above), this bird-specific ‘recalibration’ of temperature did not affect any of the quantitative analysis reported, only the temperatures reported in the figures (e.g. Figure 1G).

### Effect of HVC cooling on EP and IP amplitude (Figure S5)

The maximum depth of each IP rendition was calculated as the 1^st^ percentile of the pressure values during that rendition. The maximum amplitude of each EP rendition was calculated as the 99^th^ percentile of EP pressure values. For each identified syllable, the peak EP pressure values were normalized by the average EP peak amplitude in the control condition (normal brain temperature), thus giving the fractional deviation from control condition for each EP. The slope of a line fit to the set of fractional deviations as a function of temperature yielded the fractional amplitude change per degree C. The same procedure was repeated for IPs. Positive slopes correspond to a decrease in magnitude of EPs and IPs with decreasing temperatures.

### Fractional stretch of IPs, gaps, EPs, and syllables (Figure 2)

**Figure 2:**
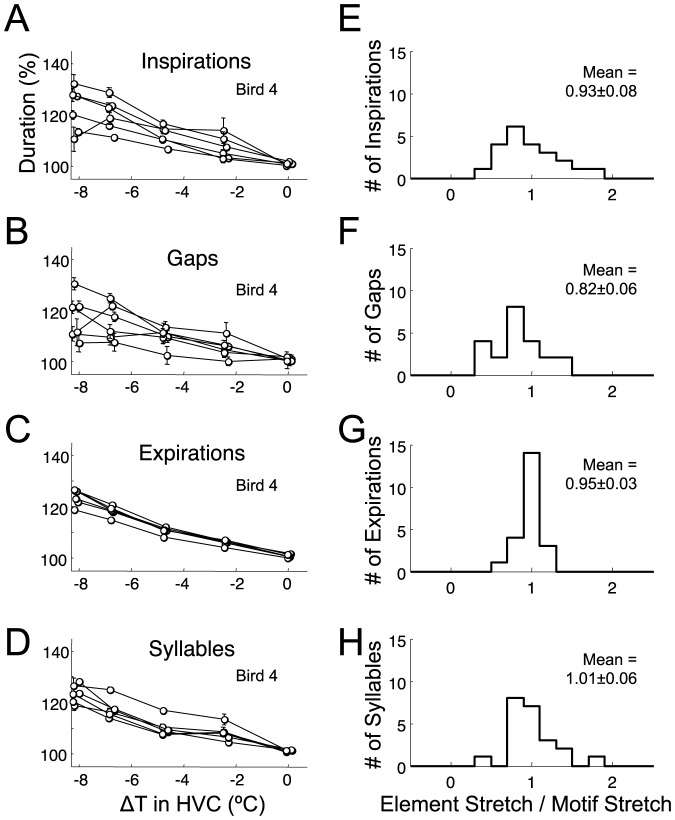
Respiratory and vocal events are lengthened equally by HVC cooling. A–D) Average duration of all IPs, gaps, EPs, and syllables in Bird 4 at different HVC temperatures as a percentage of their average duration at the control temperature (a slight horizontal jitter was added to all data points to prevent overlap of error bars; error bars in all figures indicate s.e.m. except where otherwise noted). E–H) Histogram of temperature-dependent stretch of all IPs (n = 22), gaps (n = 22), EPs (n = 22), and syllables (n = 22), normalized by the overall stretch of the song motif (n = 5 birds).

The fractional stretch of IPs, gaps, EPs, and syllables was calculated as described for EPs here: for each identified EP, the average EP duration at control HVC temperature (normal body temperature) was calculated and all EP durations were normalized by this value. In Figure 2C, the average normalized duration in each temperature bin is reported for each EP in Bird 4. A line was fit to all normalized EP durations as a function of temperature, and the slope of this line (in percent per degree) was divided by motif stretch (previous section) and reported in Figure 2G as a histogram over all EPs in all birds.

### Analysis of uniformity of EP stretch (Figure 3)

**Figure 3:**
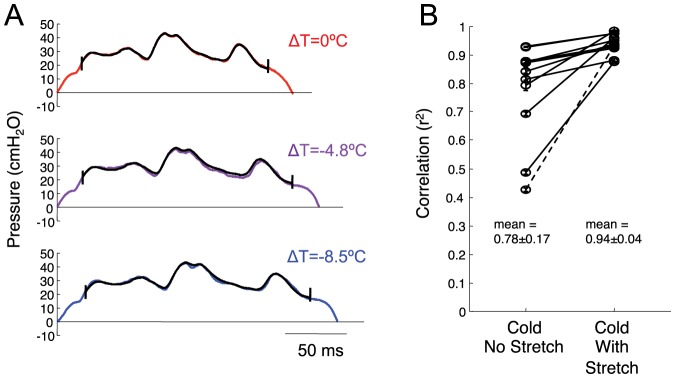
EPs are stretched uniformly by HVC cooling. A) Example renditions of an EP (Bird 4, syllable ‘c’) recorded at three different HVC temperatures (red is control, blue is coldest. The template waveform (black) is the average EP pressure waveform at the control temperature. To assess the uniformity of EP stretch, the template EP is linearly stretched to be the same length as each EP rendition and then temporally shifted to maximize its correlation with that EP. The first and last 10% percent of template and EP are excluded from the analysis. B) Average goodness-of-fit (correlation coefficient) of the template with EP renditions from the cold HVC condition with no stretch of the template (left, n = 11 identified EPs). Average goodness-of-fit when the template EP was uniformly stretched to be same length as each EP rendition before fitting (right, dashed line corresponds to the EP shown in panel A).

Eleven syllables were selected on the basis of having a complex pattern of pressure fluctuations within the syllable (selected syllables are marked with a ‘*’ in Figure S2). A template of the pressure waveform was calculated as the time average of the 100 EPs with durations closest to the median EP duration. The template EP was fit to individual EP renditions across all temperature conditions by several different methods and the goodness-of-fit was compared. The first method finds the optimal temporal shift of the template to maximize its correlation with the EP being analyzed. In the second method, each template EP is first uniformly stretched to be the same length as the EP rendition being analyzed, and then the optimal temporal shift is determined. Both of these fits involved one degree of freedom. If the increased duration of EPs under HVC cooling were due to a uniform stretch of the EP, then the second method should produce a much better fit between the template and the EPs produced with cooled HVC. On the other hand, if the increased EP duration were due to an extended period of positive pressure at the end of the EP, without changing the pressure waveform earlier in the EP, then the second method should produce a worse fit with the cooled EP.

Stretched templates were generated by linear interpolation of the original. Fitting was done by simply computing the correlation for all temporal shifts (resolution 1 ms) and choosing the maximum. For the fitting procedure, the first and last 10% of the template EP and the analyzed EP were removed to prevent the initial rising and falling portion of the EP from dominating the calculation. The fitting procedure allowed the template to shift past the beginning or end of each EP by 15%, but the correlation was carried out only on overlapping parts of the waveforms with no penalty for overhangs. The optimal shift and maximum correlation (r-squared, goodness-of-fit) were recorded for each EP rendition at both the control and the coolest HVC temperature. A minimum of 10 EP renditions were analyzed in each temperature condition (mean number of EPs: 262 control, 298 cooled). For each syllable, a paired t-test of the r^2^ values was used to compare the goodness-of-fit of the template to all EPs recorded in cooled HVC conditions for the two fitting procedures.

A third method of fitting was used to compare an optimized fit of the template to cooled and control EPs. The fit was carried out by simultaneously optimizing the amount of linear stretch of the template and the temporal shift to maximize correlation. Again, the correlation was carried out only on overlapping parts of the waveforms, with no penalty for overhangs. A comparison of goodness-of-fit was made to assess the extent to which a linear stretch captures the temperature-dependent variations in waveform. To assess the linearity of stretch, two additional metrics were computed: 1) The ratio of the optimal linear stretch of the template (determined from the fitting algorithm) to the overall fractional increase in EP duration, expressed as a percentage of the template duration; and 2) the time difference between the midpoint of the sample waveform and the midpoint of the optimally stretched and shifted template. The latter time difference was also expressed as a percentage of the total template duration. The slope of these quantities as a function of temperature was determined from a least-squares linear fit.

### Time-binned average IPs (Figure 4A and 5A)

**Figure 4:**
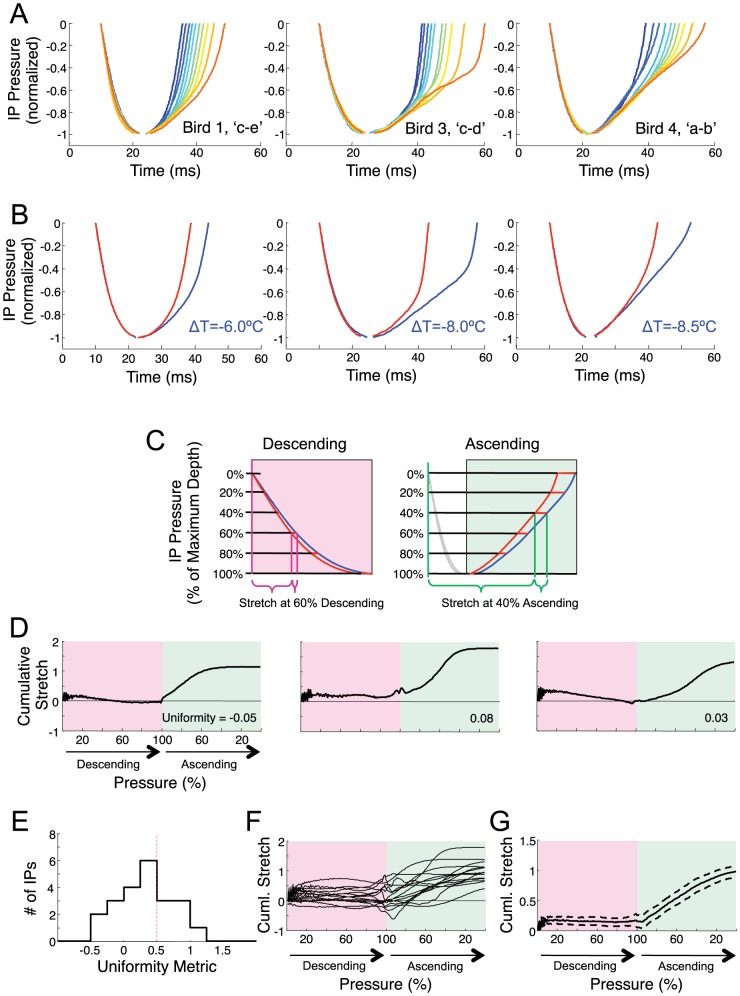
Most IPs are stretched nonuniformly by HVC cooling. A) Examples of IP waveforms of different duration from three identified gaps (Bird 1 ‘c–e’; Bird 3 ‘c–d’; Bird 4 ‘a–b’). Each trace is an average of a minimum of 10 individual IPs grouped by duration (see Methods). Different traces represent IPs sorted from shortest (blue) to longest (red), in different percentiles of the distribution of IP durations. B) Average IP waveforms recorded at control temperature (red) and at maximal HVC cooling (blue), for the IPs shown in part A. C) Illustration of how cumulative stretch is computed: the time it takes the IP to reach a certain percentage of its maximum depth (from IP onset) is measured at each temperature condition, and the temperature-dependence of this time-to-threshold is calculated. This process is repeated for all percentages on both the descending and ascending phase of the IP, and these values are normalized by the bird's motif stretch to produce a ‘cumulative stretch’ curve. Note that, for perfectly uniform stretch, the cumulative stretch curve would be constant at all points on the IP, and would be a flat line. D) The cumulative stretch curve for each IP shown in part A. These IPs exhibited a highly non-uniform stretch, with little temperature dependence during the descending phase of the IP. E) Histogram of a uniformity metric for all IPs, given by the ratio of the temperature-dependent stretch of the descending phase of the IP (measured just before the pressure minimum), to the total stretch of the IP. F) Cumulative stretch curve for each IP with a uniformity metric less than 0.5. G) Mean and standard deviation of the cumulative stretch curves in panel F.

**Figure 5:**
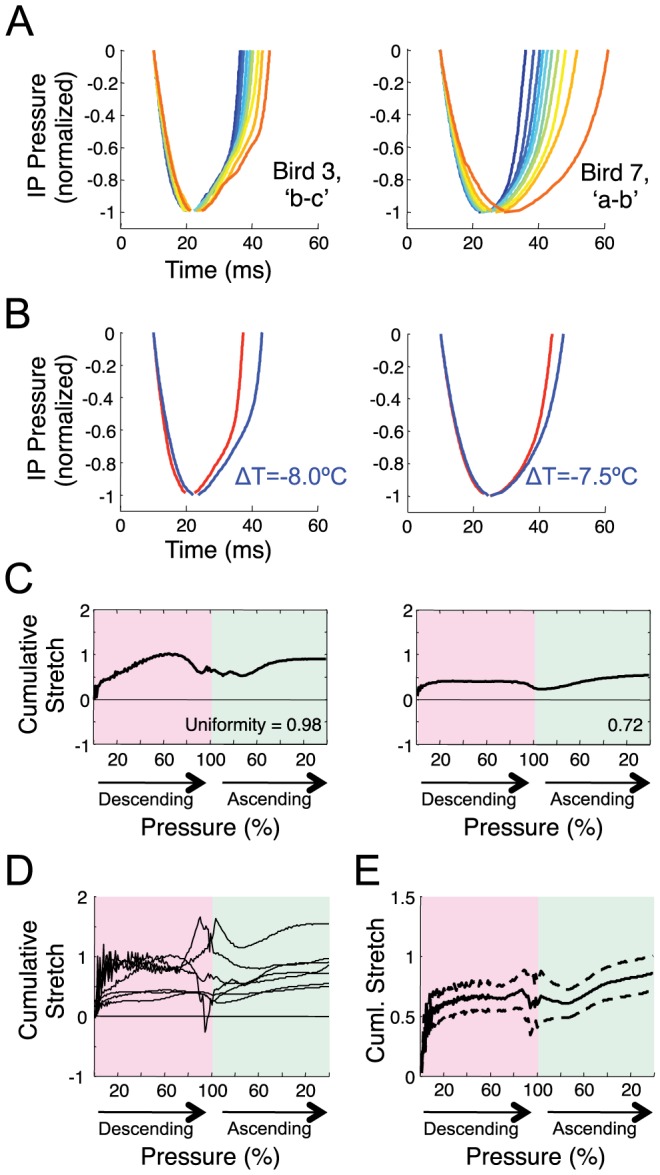
Some IPs are stretched uniformly by HVC cooling. A) Examples of IPs that show uniform stretch (Bird 3 ‘b–c’; Bird 7 ‘a–b’). Each trace is an average of a minimum of 10 individual IPs grouped by duration (see Methods) from shortest (blue) to longest (red). B) Average IP waveforms recorded at control temperature (red) and maximal HVC cooling (blue) for the IPs shown in part A. C) The cumulative stretch curve of the IPs shown in part A. These IPs exhibited a near uniform stretch throughout descending and ascending phases. D) Cumulative stretch curves for all IPs with a uniformity greater than 0.5. E) Mean and standard deviation of these cumulative stretch curves.

The analysis of IP waveforms under HVC cooling was carried out using a different approach than for EP waveforms. Unlike EPs, most IPs consist of a single ‘dip’ with a monotonically decreasing initial phase and a monotonically increasing terminal phase, allowing a detailed quantitative analysis of waveform shape. This analysis consisted of duration-binned averaging, temperature-binned averaging, and cumulative stretch analysis.

Duration-binned average IP traces were computed to show the relation between IP shape and IP duration. For each identified syllable transition, IPs were sorted by duration, and IPs were selected around 10 different percentiles centered at 4%, 14%, 24%, 34%, 44%, 54%, 64%, 74%, 84%, and 94%. In each case IPs were selected in a range of −2% to +2% around the centers. Each IP trace was normalized by its maximum depth and the normalized traces were averaged to produce the traces in Figure 4A. The averaging was done along the time axis (meaning that time values at given pressure were averaged together), rather than along the pressure axis, since this produced a more reliable estimate of mean IP shape. By necessity, the temporal averaging was carried out separately for the descending and ascending phases of the IP.

### Temperature-binned average IPs (Figure 4B and 5B)

Average IP traces were also computed to show the IP shape at different HVC temperatures. For each identified syllable transition, IPs were normalized and then averaged separately for the control condition and the maximal HVC cooling condition. As in the previous section, averaging was done along the time axis.

### Cumulative stretch analysis of IPs (Figure 4D and 5C)

In order to quantify the precise effect of HVC cooling on IP waveform, we carried out a ‘cumulative stretch analysis’. For each IP rendition in each temperature condition, the time at which the IP crosses a set of amplitude thresholds was measured relative to IP onset. This was done separately during the initial descending phase of the IP and during the final ascending phase of the IP (Figure 4C). In the descending phase, for example, for a threshold of 50%, the time from IP onset to the time at which the pressure crosses 50% of its minimum value was recorded (Figure S6A–C). These times were calculated for all threshold values from 0% to 100% in steps of 1%. All threshold-crossing times were then normalized by the average time to the threshold crossing in the control condition.

To determine the cumulative temperature dependence of the IP waveform, a line was fit to the threshold crossing times as a function of temperature, for each choice of threshold. In the descending phase for example, a line was fit to the 50% descending threshold crossing times (normalized by the 50% crossing time at control temperature), as a function of temperature (Figure S6D–F); the slope of this line was normalized by the motif stretch for the respective bird to give the cumulative stretch at the 50% threshold. This was repeated at all threshold values from 0 to 100% on the descending phase, and then for all threshold values from 100% to 0% on the ascending phase to create the entire cumulative stretch curve (Figure S6G; Figure 4D).

The cumulative stretch analysis determines temperature-dependent changes in the time from IP onset until the pressure crosses a certain threshold. We also carried out a ‘local stretch analysis’ to examine local temperature-dependent changes in waveform slope. Local slope was estimated based on the time it takes the IP pressure to change by 10 percent of the maximum pressure from each point on the descending and rising phases (i.e. from the 40% point to the 50% point). The details of this procedure are shown in Figure S7 and the results are shown in Figure S11.

### Uniformity metric (Figure 4E)

A metric of uniformity of stretch was computed by comparing two quantities: 1) the temperature-dependent elongation of the time from IP onset to near the IP minimum, and 2) the change of the entire IP duration. To produce a robust estimate of the stretch near the peak, the uniformity metric was averaged between 75% and 85% thresholds on descending phase. The uniformity metric is given by the ratio of these quantities, giving a value of 1 if the stretch of the time-to-minimum is the same as the total stretch, and 0 if the time-to-minimum has no temperature dependence.

The histogram of uniformity metric for IP stretch during natural variability shown in Figure S12C) is shown excluding two large outliers (values: 3.1 and 3.7). These large values of uniformity were caused by a slight bump in the shape of these two particular IPs near the minimum, causing the linear stretch analysis to exhibit a large peak near 100% (see Figure S13C). Because the mean and standard error are sensitive to large outliers, these measures of the uniformity metric for IP stretch during natural variability are quoted in the text (mean = 0.31±0.10) with these unrepresentative outliers removed. All other statistics regarding uniformity metric were computed including these two IPs.

### Effect of tracheosyringeal nerve section on EP waveform and EP stretch (Figure S8)

In two birds (Bird 4 and Bird 7), ts-nerve sections were performed after intact-ts-nerve experiments were completed. The effect of ts-nerve section on EP waveform was analyzed by computing the average cross correlation between 20 randomly selected pairs of pre-lesion and post-lesion EPs for each syllable generated by these birds. The effect of cooling on EP waveforms following ts-nerve section was assessed using methods analogous to those used in Figure 3B. A template pressure waveform was computed based on syllables produced following ts-nerve section in the control temperature condition. This template was fit to syllables produced in control and cold conditions using the two methods described above (n = 5 syllables in two birds).

## Results

### Effect of HVC cooling on inspiratory and expiratory pulse durations

We set out to examine the role of cortical nucleus HVC in the timing of respiratory song components and coordination of respiration with song syllables. We simultaneously recorded air sac pressure and song vocalizations under different HVC temperature conditions (n = 5 birds). Song syllables were labeled (‘a’, ‘b’, ‘c’) and individual gaps were identified by the surrounding syllables (e.g. ‘a–b’, ‘b–c’, etc., Figure S3). Motifs were identified as the most probable sequence of syllables. As previously reported [9], we found that song motifs were slowed at colder HVC temperatures (Figure 1F, 20–30% slowing at maximum cooling of −8.5°C), and that individual syllables and gaps slowed by an amount similar to song motifs. The ratio of syllable and gap stretch to that of the entire song motif was found to be 1.01±0.06 and 0.82±0.06, respectively (gap stretch significantly smaller than 1, p<0.01 t-test, n = 22 syllables, n = 22 gaps, ±s.e.m. except where otherwise noted).

Respiratory patterns were also slowed by HVC cooling (Figure 1F and G); expiratory pressure pulses (EPs) and inspiratory pressure pulses (IPs) increased in duration by an amount similar to song motifs (Figure 2, factor of 0.95±0.03 for EPs and 0.93±0.08 for IPs, not significantly different from one, p>0.05 for both). HVC cooling produced a small but significant decrease in EP amplitude by 0.51±0.12%/°C (p<0.01, Figure S5), resulting in an average 3.0±2.3% decrease at the lowest temperatures. In contrast, on average, there was no significant change of IP depth with HVC cooling (−0.095±0.22%/°C, p>0.5, t-test except where otherwise noted). The average decrease in IP depth at maximal cooling was not significantly different from zero (0.15±2.5%, p>0.5).

### Effect of HVC temperature change on directed and undirected song

Changes in HVC temperature affected the duration of EPs and IPs during both directed and undirected song. Between the two social contexts, there was no significant difference in the temperature dependent stretch of EPs (n = 14, p>0.1, paired t-test, Figure S9) nor of IPs (n = 15, p>0.5, paired t-test). Consistent with earlier reports, we found that, on average, EP durations were significantly shorter in directed song than in undirected song (1.73±0.42% shorter, n = 14, p<0.001, paired t-test), and that, on average, IP durations were not significantly different (Figure S10A and B, 0.30±0.88% longer, n = 15, p>0.5). We also note that, while IP durations were not affected by social context *on average*, many identified IPs exhibited significant lengthening or shortening between directed and undirected song (n = 7/15, Figure S10C and D). Overall, the effect of social context on EP and IP durations was smaller than the effect of manipulating HVC temperature (Figure S10A and B).

### Expiratory pressure pulses (EPs) were stretched uniformly by HVC cooling

We wondered about the mechanism underlying the increased duration of EPs at colder HVC temperatures. Do EPs undergo a uniform linear stretch, or is the increased duration due to a prolongation of positive pressures at either the beginning or end of the EP? To distinguish these hypotheses, we examined EPs with a complex stereotyped pattern of pressure fluctuations within the syllable, and found that these EPs appeared to be stretched uniformly by HVC cooling (Figure 3A). To quantify the uniformity of EP stretch, for each complex EP we used the average pattern of pressure fluctuations (EP waveform) in the control condition as a template (Figure 3A, top) to compare with EP waveforms under cooled conditions (Figure 3A, middle and bottom).

We compared how well the template patterns could be made to fit EPs produced under cooled HVC conditions using two fitting methods, corresponding to the two hypotheses described above (see methods). Attempting to fit the template with the cooled EP waveforms using only a temporal shift of the template did not yield particularly good fits (Figure 3B, mean r^2^ = 0.78±0.17 s.d. for all complex EPs, n = 11). However, by first uniformly stretching the template EP to have the same duration as each EP rendition before fitting, the fit of the template to the cooled EPs was significantly better for all 11 EPs examined (Figure 3B, paired one-sided t-test, p<0.001 for each EP, mean r^2^ = 0.94±0.04 s.d. over all EPs).

As a further test of the uniformity of EP stretch, we compared the fit in the cooled and control conditions when the amount of linear stretch of the template was optimized to produce the best fit, rather than being fixed (see Methods). The stretch-optimized fit of the template to the cooled EPs (r^2^ = 0.95±0.03) was nearly as good as the fit to the control EPs (r^2^ = 0.98±0.02), suggesting that linear stretch captures the majority of the waveform changes between EPs in cooled and control conditions. Furthermore, the ratio of the optimal template stretch to the fractional change in EP duration was not significantly different from one (1.001±0.003, p = .70) over the entire range of HVC temperatures (slope as a function of temperature: −0.10±0.08%/°C, not significantly different from zero, p = 0.25). The shift between the midpoint of the stretched template and the midpoint of the fitted EP waveform, expressed as a percentage of the template duration, was less than 1.0% over the entire range of temperatures (−0.76±0.20% in the cold condition, slope as a function of temperature 0.15±0.03%/°C, p<0.001). Both of these findings are consistent with the hypothesis that EPs are stretched uniformly by HVC cooling, and are not consistent with the hypothesis that changes in the duration of EPs are produced by earlier or later truncation of either end of an otherwise unchanging EP waveform.

Are the patterned fluctuations of pressure during EPs produced by direct forebrain control of pressure, or are these fluctuations a byproduct of modulation of airflow by the syrinx? To answer this question we carried out bilateral transection of the tracheosyringeal nerve (Figure 1A) after previously recording air sac pressure during singing (n = 2 birds, see Methods). In both birds, bilateral transection of the ts-nerve left the respiratory pattern of EPs and IPs intact (Figure S8). In syllables with complex patterns of pressure fluctuations, the waveform was largely unaffected by nerve transection (n = 5 syllables, correlation between pre- and post-lesion EPs, r^2^ = 0.89±0.04, not significantly different from the correlation between 20 pairs of post-transection EPs, r^2^ = 0.94±0.03, p>0.3, paired t-test, n = 5 syllables). After transection, the EPs increased in duration with HVC cooling by the same amount as the motif (1.10±0.06 normalized to motif stretch, not significantly different from one, p>0.1, Figure S8C and F), and the template fitting procedure described above revealed that the EP pressure waveforms were well modeled by a uniform stretch under HVC cooling (Figure S8G, n = 5 syllables, template fit with stretch, mean, r^2^ = 0.91±0.06 s.d., template fit without stretch, mean r^2^ = 0.64±0.29, p<0.05 for each EP, paired one-sided t-test, see Figure S8 for further analysis). This result suggests that HVC directly controls the timing of expiratory patterns during song syllables.

### Most IPs are stretched non-uniformly by HVC cooling

Many aspects of song timing appear to slow uniformly in concert with the duration of the song motif — subsyllabic structure, syllable and gap durations (Long and Fee, 2008), EP and IP duration (Figure 2), and patterns of pressure fluctuations within EPs (Figure 3). We next examined if the same was true of the pressure waveforms within IPs. Interestingly, by examining IPs of different duration, we found numerous examples of gaps in which IP waveforms appeared not to be elongated by uniform stretch. In these instances, shorter renditions of these IPs appeared to us to resemble truncated versions of longer renditions, prematurely interrupted by a rapid transition from inspiratory to expiratory pressure (Figure 4A). We will address this interpretation of changes in IP shape more completely in the discussion.

To more fully quantify how IP pressure waveforms changed as a function of duration, we analyzed the temperature dependence of IP waveforms (Figure 4B) using a ‘cumulative stretch analysis’ (see Methods and Figure S6). This analysis examines changes in the time it takes, relative to the IP onset, for the inspiratory pressure to reach any given percentage of the minimum pressure, for both the descending and ascending phase (Figure 4C). If the IP waveform stretched uniformly with HVC cooling, this metric would be constant throughout the IP, whereas for a truncated IP, it would be close to zero except near the end of the IP. This analysis confirms that, for the examples shown in Figure 4A, the pressure waveform of the IP shows little temperature dependence until the last third of the IP (Figure 4D). Similar results were obtained with a ‘local stretch metric’ that quantifies local changes in waveform slope (Figure S7 and S11).

We quantified the uniformity of the IP stretch using a metric given by the cumulative stretch near the minimum of the IP (i.e. temperature dependence of the descending phase) divided by the total stretch of the IP (see Methods). The metric has a value of 1.0 if these quantities are equal, consistent with a uniform stretch. The metric is zero if the time of the pressure minimum has no temperature dependence. While we found a broad distribution of IP uniformity metrics across all gaps (Figure 4E), the median uniformity metric was 0.38 (mean 0.34±0.42, s.d.). In fact, the majority of IPs showed a uniformity metric less than 0.5 (Figure 4E–G, n = 15/22 IPs from identified gaps in 5 birds), suggesting that most gaps contain an IP exhibiting non-uniform variations in duration. However, not all IPs exhibited such non-uniform stretch behavior; a significant number of identified gaps in which IPs of different durations appeared to be linearly stretched versions of each other (Figure 5A and B, n = 7/22 IPs had a uniformity metric greater than 0.5). For these IPs, the cumulative stretch analysis revealed a more constant stretch metric throughout the descending and ascending phases of the IP (Figure 5C–E).

We next investigated whether the non-uniform stretch of IPs that resulted from HVC cooling was also observable in natural variation of the duration of IPs. We therefore carried out a cumulative stretch analysis on IP waveforms acquired only at control HVC temperature. The analysis was similar to that described above, but rather than computing the cumulative stretch as a function of HVC temperature, we computed cumulative stretch as a function of IP duration (Figure S12 and S13). The median uniformity metric was 0.34 (mean = 0.31±0.10, n = 22; Figure S12C), and 68% of IPs exhibited a uniformity metric less than 0.5 (n = 15/22). Furthermore, 12 of the 15 IPs that exhibited the most non-uniform stretch under HVC cooling (uniformity<0.5) also showed non-uniform stretch during natural variations (uniformity<0.5). This suggests that the non-uniform stretch of IP durations we observe is not simply an artifact of the HVC cooling, but reflects a process occurring naturally during singing.

**Figure 6:**
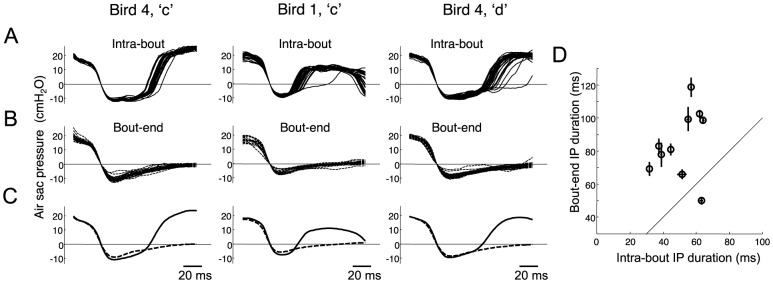
IPs are longer at bout offset. A) Examples of identified IPs produced within a song bout, where there was a following syllable. B) Examples of the same IPs produced at bout-end, where there was no following syllable. C) Average of the intra-bout IPs (solid) and bout-end IPs (dashed). D) Scatter plot of duration of IPs following bout-ending syllable renditions versus intra-bout syllable renditions for the 10 syllables that occurred at bout-ends with sufficient frequency to be analyzed.

In light of the possible interpretation that IPs are truncated by the EP at the onset of the next syllable, we wondered if IPs that occurred at the ends of bouts, with no following syllable, were longer in duration. We found that in most cases, these bout-ending IPs resembled untruncated versions of intra-bout IPs (Figure 6A–C), Of the 22 syllables in the dataset, only 10 occurred at bout ends sufficiently often to analyze quantitatively (>20 renditions). Among those, 9 produced IPs that were significantly longer when the syllable was at a bout end than within a bout (Figure 6D, p<0.05 for 9 syllables), and the mean IP duration over all IPs was significantly longer at bout ends than during bouts (bout end, 85±6.5 ms; intra-bout, 51±3.7 ms, p<10^−3^ paired t-test, n = 10).

### Analysis of the coordination between gaps and IPs

**Figure 7:**
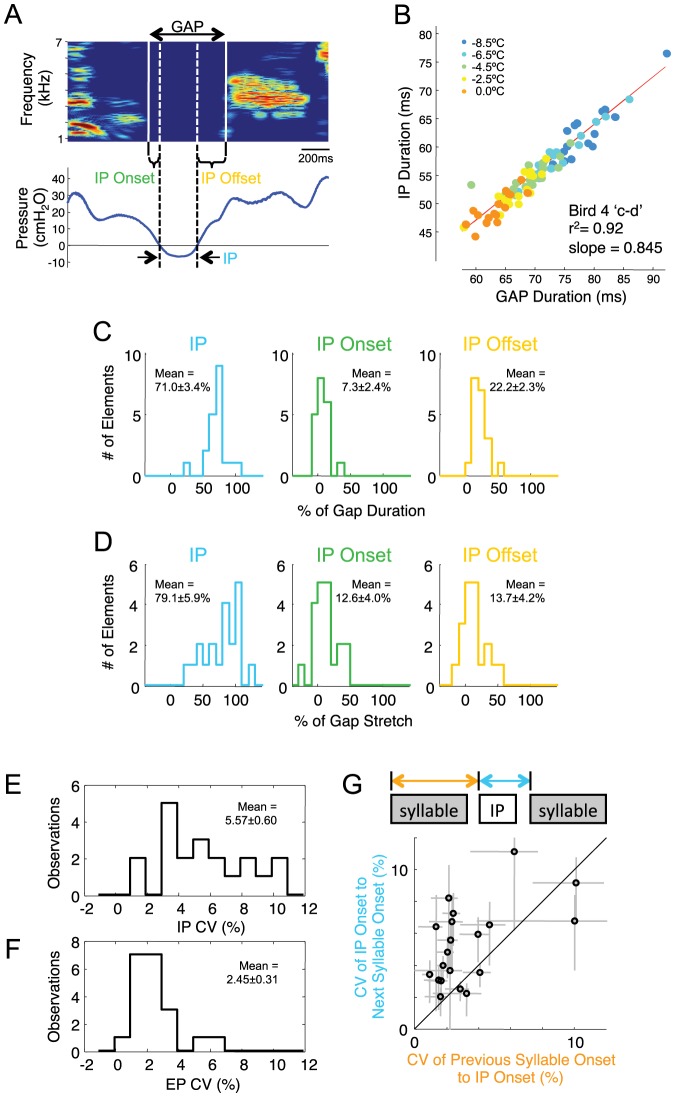
Analysis of respiratory-vocal coordination: HVC cooling and natural variability. A) Song spectrogram of two syllables showing the silent gap between them, and a simultaneous recording of thoracic air sac pressure. Three distinct components of the gap are identified: the IP onset period (between the end of the syllable and the start of the IP), the IP itself, and the IP offset period (between the offset of the IP and the onset of the syllable). B) Scatter plot of IP duration versus gap duration for 100 renditions of one gap (Bird 4 ‘c–d’) over all temperature conditions (orange, warm; blue, cold). C) Histograms of the fraction of the gap occupied by the three gap components (IP, left; IP onset period, middle; IP offset period, right). Each identified gap (e.g. ‘a–b’, ‘b–c’) is analyzed separately (n = 20 gaps, from directed song only). D) Individual contribution of IP, IP onset period, and IP offset period to the overall temperature-dependent stretch of gaps for all syllable transitions. E) Coefficient of variation (CV) of IP durations (n = 20 identified gaps) and F) CV of EP durations (n = 20 syllables, directed song only). IPs show significantly higher fractional variability than EPs (CV of IPs, 5.57±0.60%; CV of EPs, 2.45±0.31%, p<0.001). G) Scatter plot comparing, for each IP, the CV of the interval from IP onset to both the proceding and the following syllable onset (n = 20 gaps, directed song only).

Earlier studies of respiratory patterns in adult song have suggested that respiration is tightly coordinated with vocal patterns [42]. However, the non-uniform changes in IP shape, and the apparent truncation of many IPs, led us to examine at a more quantitative level how the beginning and end of IPs relate in time to the surrounding syllable offsets and onsets. The fact that gaps stretch by a similar amount as IPs with HVC cooling (Figure 2) suggests that variations in gap duration may be highly correlated with variations in IP duration. Indeed, this was the case for all gaps analyzed (Figure 7A and B, mean r^2^ = 0.82±0.03, mean slope = 0.75±0.03), suggesting that prolonged IPs are associated with a delayed onset of the next syllable.

To refine this analysis, we explicitly analyzed the duration of three separate gap components (Figure 7A): 1) the interval from syllable-offset to the onset of the IP (IP onset period), 2) the IP itself and 3) the interval between the offset of the IP and the onset of the syllable that terminates the gap (IP offset period). IPs had a mean duration of 35.2±2.4 ms, which on average made up 71.0±3.4% of the total gap duration (Figure 7C, left). In contrast, IP onset periods were very brief (mean IP onset period, 4.24±1.7 ms) and occupied on average only 7.3±2.4% of the total gap duration (Figure 7C, middle). For 9 IPs, a significant fraction of syllable renditions extended past the onset of the IP, such that 5 IPs had a negative mean IP onset period. Thus, gaps are largely filled by IPs, and IP onsets typically occur immediately after or nearly simultaneously with syllable offsets. Finally, IP offset periods were typically longer than IP onset periods (11.2±1.4 ms), occupying on average 22.2±2.3% of the total gap duration (Figure 7C, right).

As noted above, the strong correlation between variations in gap duration and IP duration suggests that syllable boundaries are tightly coordinated with the beginning and ends of IPs. This is borne out by the small absolute timing jitter of IP onset and offset periods (1.32±0.14 ms and 1.13±0.12 ms RMS jitter, respectively, see Table 1 for further quantification). These are not significantly different from each other (p = 0.33), but both of these are significantly smaller than the jitter in the interval from IP onsets to the beginning of the following syllable (2.4±0.26 ms, p<10^−4^ paired t-test) and the jitter in IP durations (1.90±0.24 ms, p<0.002, paired t-test). Note that a substantial fraction of the jitter observed in IP onset and offset period durations may come from measurement error in syllable onsets and offset times due to acoustic fluctuations, rather than variability in the timing of the underlying neural activity. RMS noise in syllable onset times is likely in the range of 0.5 to 1 ms, and even higher for syllable offsets [8], [43].

**Table 1:** Shown are the average duration and root-mean square jitter (standard deviation) of each measure of respiratory/vocal coordination.

Feature	Average Duration (ms)	Absolute Jitter (ms)	Fractional Jitter (c.v. %)
IP	35.2±2.4	1.90±0.24	5.6±0.6
EP	126±12.8	2.68±0.24	2.4±0.3
Gap	50.4±3.2	2.80±0.30	5.6±0.5
Syllable	107±13.7	3.03±0.30	3.8±0.7
IP onset period	4.24±1.7	1.32±0.14	**−8.2±20.6**
IP offset period	11.2±1.4	1.13±0.12	**40.0±29.0**
IP onset to next syllable onset	46.4±2.6	2.4±0.28	5.3±0.6
Syllable offset to IP offset	39.6±2.7	2.9±0.45	7.5±1.2
Syllable onset to next IP onset	110±13.4	2.8±0.25	3.4±0.6

Both absolute jitter (ms) and fractional jitter (% c.v.) are shown. Error bars indicate s.e.m for each quantity. IP onset period is the period from the end of each syllable to the onset of the following IP. IP offset period is the period from the offset of the IP to the onset of the following syllable. The last item (syllable onset to next IP onset) is the quantity shown in yellow in Figure 7G. Note that the average IP onset and IP offset periods are very short. In fact, for some gaps the IP onset period is negative, meaning that the syllable extends slightly into the IP. Because of this, the measure of fractional jitter (text in bold) is highly unreliable and should not be considered.

To gain further insight into how the coordination between IPs and syllables is regulated by HVC, we examined the effect of HVC cooling on these three gap components. To do this, we computed the temperature-dependent change in duration (in milliseconds) of each component, divided by the temperature-dependent change in gap duration (in milliseconds). We found that the change in IP duration accounted for 79.1±5.9% of cooling-induced gap stretch (Figure 7D, left), not significantly different from the fraction of the gap duration occupied by the IP (p>0.2). Stretch of IP onset periods contributed 12.6±4.0% of the total gap stretch, and IP offset periods contributed 13.7±4.2% to the gap stretch, neither of which was significantly different from their respective contributions to gap duration (Figure 7D middle and right, p>0.05 for both comparisons). These findings suggest that HVC plays a role in controlling the onset and offset of all gap components.

Earlier studies have suggested that gap durations are intrinsically more variable than syllable durations, and may therefore involve an additional ‘noisy’ mechanism [44]. By analogy, we examined the relation between IP and EP variability. We calculated the coefficient of variation (CV) of IP durations within each identified gap and of EP durations for each syllable, defined as the standard deviation of durations across different renditions, normalized by average duration [44]. IPs exhibited substantially larger CVs than did EPs (Figure 7E and F; IP, mean CV = 5.57±0.60%; EP, mean CV = 2.45±0.31%; p<10^−5^). Consistent with this, the jitter (CV) of the interval from IP to following syllable was on average 2.0 times larger than the jitter of the interval from IP onset to the previous syllable onset (Figure 7G, p<0.001, paired t-test). We will interpret these findings in terms of a hypothesis for HVC function in which syllables and respiratory patterns are controlled by multiple synaptically-connected chains in HVC [9], [32].

## Discussion

We have addressed the question of how inspiratory and expiratory pressure pulses are coordinated with syllables and gaps in adult zebra finch song. To quantitatively analyze the relation between respiratory and vocal gestures, we simultaneously recorded thoracic air sac pressure and song vocalizations. Localized cooling of forebrain premotor nucleus HVC was used to specifically analyze the role of this nucleus in respiratory-vocal coordination.

### HVC cooling produces a uniform stretch of EPs and non-uniform stretch of most IPs

We found that cooling of HVC not only causes slowing of syllables and gaps, as previously described [9], but it also increases the duration of expiratory pressure pulses (EPs) and inspiratory pressure pulses (IPs). The lengthening of EPs was associated with a uniform stretch of the detailed pattern of pressure fluctuations within the syllable. It was previously reported that HVC cooling causes a uniform slowing of the fine acoustic structure within syllables [9], suggesting that, during HVC cooling, EP pressure fluctuations remain precisely coordinated with the acoustic changes within syllables. In addition, the pattern of pressure fluctuations during EPs was largely unchanged by transection of the tracheosyringeal nerve, and the pressure fluctuations within EPs were uniformly stretched by HVC cooling even following nerve transection. These observations are consistent with previous findings that pressure fluctuations are not simply a result of modulation of airflow by the syrinx, but are actively driven by central control of respiratory musculature [16].

Just as for EPs, we found that HVC cooling increased IP durations, suggesting that biophysical processes in HVC control the time between IP onsets and offsets. However, in contrast to EPs, we found that HVC cooling produced a non-uniformly stretched version of the IPs in the majority of identified gaps. In these cases, we observed little change in the shape of the early part of the IP, but pronounced elongation of the later part. This was true not only for variations in IP duration produced by HVC cooling, but also for natural variations in IP duration.

### The relation between respiration and sparse burst sequences in HVC

How can we explain the observation that EPs stretch uniformly, while IPs stretch non-uniformly? We will examine several possible interpretations of this observation in the framework of a model of sequence generation in which the vocal pattern is generated by a sparse sequential activation of neurons in HVC [25].

It has been proposed that the sequential activation of neurons within HVC relies on a synaptically-connected chain-like organization of groups of RA-projecting HVC neurons [9], [30]–[32]. In this model, HVC_(RA)_ neurons both activate each other in sequence and also activate a complex sequence of spike bursts in downstream nucleus RA [8]. The activity in RA converges downstream to generate a precise continuous sequence of activity in syringeal motor neurons and muscles [34]. In the context of this model, the finding that HVC cooling results in a uniform stretch of the pressure pattern of expiratory pulses is consistent with the idea that expiratory pressure during singing is continuously controlled, on a moment-to-moment basis, by HVC. For example, at any moment in time, HVC could drive activity in the subset of RA neurons that project to the avian homologue of the ventral respiratory group [13], which would then produce a certain level of activation of the expiratory musculature. A few milliseconds later (∼6 ms), a different set of HVC neurons would become active, which would then produce a different level of downstream activity and a different expiratory pressure. In this ‘continuous control’ model of EP generation, cooling of HVC would uniformly slow the sequential activation of HVC_(RA)_ neurons, thus producing a uniform stretching of the expiratory pressure waveforms and increasing the EP duration.

Now let us consider the role of HVC in the generation of IPs. We found that some gaps have IPs that are fairly uniformly stretched by HVC cooling, suggesting that, at least for some IPs, the pressure waveform may be controlled by HVC continuously, on a moment-to-moment basis. This could occur by a mechanism similar to that described above for EPs, perhaps via the projection from RA to inspiratory brainstem centers, such as PAm [37], [45], [46]. While uniform stretching behavior of some IPs under HVC cooling is easy to explain, the non-uniform stretch of most IPs requires that we hypothesize a more complex timing mechanism. Here we consider two possibilities.

**Figure 8:**
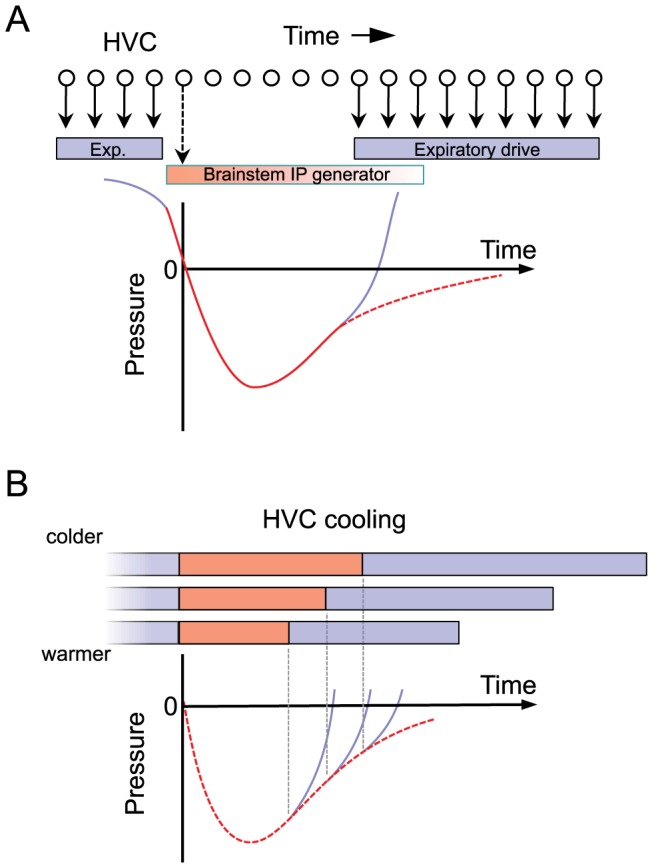
Sparse HVC sequences and a hypothesis for their control of respiratory patterns. A) HVC neurons generate a sparse sequence of activity. Each neuron is active only once per song rendition, and it has been suggested that a sub-population of these neurons is active at each moment in the song [34], [57]. Here we propose that some IPs are generated by a mechanism in which HVC initiates and terminates IPs (I/T model). In this model, HVC ‘triggers’ downstream circuitry (perhaps in the brainstem) that controls the activation and time course of inspiratory pulses. Early in the IP, HVC exerts little control on the time course the pressure waveform. At the end of the IP, HVC again takes over control of respiratory circuitry to generate the EP for the next syllable. B) This model predicts the non-uniform stretch of most IPs observed in our experiments. Slowing of the HVC chain by cooling HVC increases the interval between IP initiation and termination, thus increasing the IP duration without changing the shape of the early part of the IP. Thus, the temperature-dependent stretch of the IP waveform occurs only in the later parts of the IP. Changes in the IP waveform can be described as the earlier or later truncation of an underlying IP waveform.

First, the non-uniform stretch of some IPs was such that inspirations of different lengths appeared to be generated from an underlying long inspiratory pulse that was interrupted by a rapid transition to expiratory pressures at different times. This interpretation is consistent with the possibility that HVC simply initiates an IP at the end of a syllable, and then, prior to the next syllable, terminates the IP by driving a rapid activation of expiratory pressure (Figure 8A). In this view, which we term the ‘initiation/termination’ (I/T) model of IP generation, the pressure waveform early in the IP could be controlled by circuitry downstream of HVC (i.e. an IP generator), during which time HVC may exert little influence on the pressure. The fact that localized cooling in RA has no effect on syllable or gap timing [9] suggests to us that the IP waveform is strongly influenced by circuits downstream of RA, possibly within respiratory brain centers [13] (discussed in more detail below). More precise localization, within circuitry downstream of HVC, of the biophysical dynamics underlying IPs could be achieved with cooling in RA [9] or respiratory centers, together with simultaneous recording of air sac pressure.

The proposed I/T mechanism would explain the lengthening of IP duration with HVC cooling, as well as the fact that, for many gaps, the shape of the IP is relatively unchanged by HVC cooling until near the end of the IP (Figure 8B). Furthermore, this mechanism would imply that, in cases where the IP is not followed by another syllable, the IP would be longer. Indeed, we find that IPs at the ends of bouts, for which there is no following syllable, have a duration almost double that of IPs that occur between song syllables (Figure 6). Note that the continuous control model and the initiation/termination model of IP generation are not mutually exclusive. In fact both mechanisms can operate simultaneously, thus accounting for the broad distribution of uniformity metrics.

A second possible explanation for non-uniform IP stretch, in contrast to the I/T model, is that there could be a highly non-uniform temperature sensitivity of the sequence-generating circuitry in HVC. Specifically, one could imagine that the speed of the sequential activation of HVC bursts early in the IP is largely insensitive to temperature changes in HVC, while the sequential activation of HVC bursts in the later part of the IP is extremely sensitive to temperature changes. Thus, during HVC cooling, the descending phase of the IP would not be stretched while the latter part of the IP could exhibit large stretches, just as we have observed. While we cannot rule out this model, it postulates an unlikely complexity in the properties of the HVC sequence-generating circuit. Thus, in the absence of additional evidence to the contrary, we favor an explanation of the non-uniform stretch of IPs in terms of the I/T model.

### The role of syllable-chains in HVC

It has been suggested that the zebra finch song motif is not generated by a long, single chain, but rather by multiple chains that are linked together to produce the song syllable sequence [9], [47], [48]. Furthermore, several lines of evidence point to syllables (or sections of complex syllables) being the basic behavioral ‘units’ of the song. Bright flashes of light that interrupt singing tend to terminate the song at the ends of syllables, or at certain well-defined points within complex syllables [49]. Similarly, bilateral recordings of multiunit activity in HVC reveal brief peaks of bilateral synchronization prior to the onset of each syllable and occasionally at other times in the song, including note transitions within complex syllables [50]. These observations, together with the observation that gaps are substantially more variable in duration than syllables [44], have led to the suggestion that these syllable (or sub-syllabic) units may each be encoded by a distinct HVC module [44] or chain [9], [51]. Although several experiments have suggested that there is not a simple one-to-one relation between the hypothesized chains in HVC and syllabic structure [9], [48], [50], we interpret our observations in the context of a simplified view that distinct syllables are each encoded by a single HVC chain.

**Figure 9:**
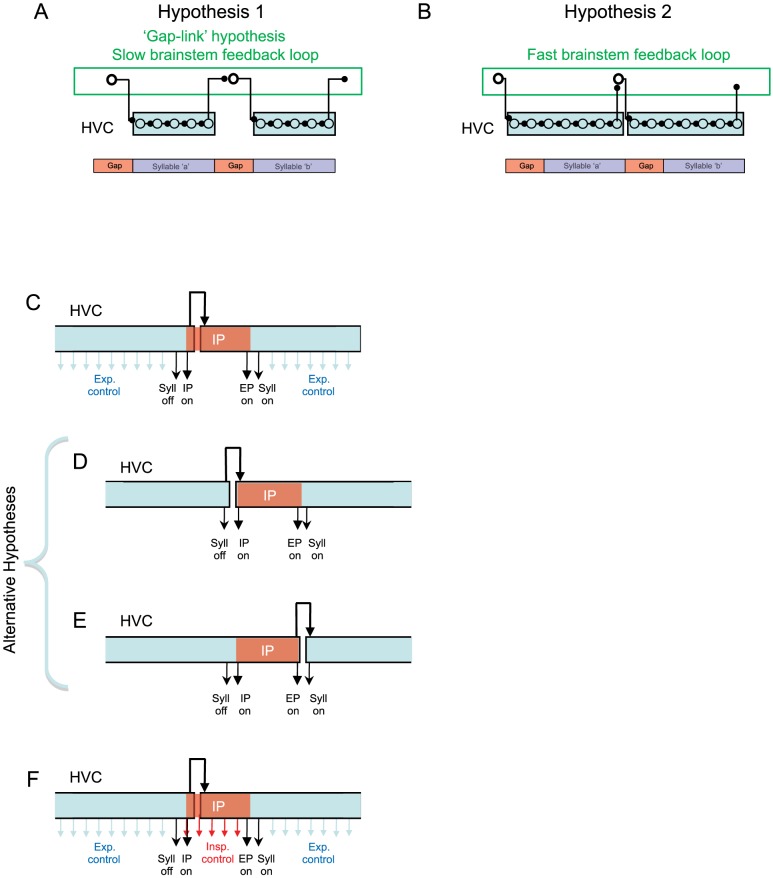
Linked-chain model of HVC and a hypothesis for its interaction with the IP generator. HVC may contain modules (light blue rectangles) of synaptically connected chains of neurons that activate each other sequentially. It has been hypothesized that each chain may code for a syllable or part thereof, and that these chains may activate each other sequentially via a bilateral brainstem feedback loop [9]. However, this sequential activation could relate to syllables and gaps in a number of ways. A) One possibility is that syllables are generated by HVC chains while gaps are produced during slow feedback through the brainstem loop (‘gap-link’ hypothesis). B) Another hypothesis in which gaps and syllables are both generated by HVC chains, and each chain is activated at the end of the previous chain by fast feedback through the brainstem loop. We argue that the model in panel A is not supported by existing data. C–E) Three different hypotheses for how IPs could be coordinated with the transition between syllable chains in HVC. C) The end of the first chain could terminate the syllable and initiate an IP. After control is transferred to the next chain by the fast feedback loop, the second chain terminates the IP and initiates the next syllable. D) An alternative hypothesis in which the first chain terminates the syllable and the second chain initiates the IP, terminates the IP and then initiates the next syllable. E) Another alternative hypothesis in which the syllable termination, IP initiation and termination are all controlled by the first chain. Previous electrophysiological studies, as well as an analysis of variability in song and respiratory timing, support the model in panel C. E) In some cases, HVC may maintain continuous direct influence the pressure waveform during the IP, thus accounting for the uniform stretch of some IPs with HVC cooling.

The fact that HVC cooling increases the intervals between syllable onsets has led to the suggestion that each syllable is activated at the end of the previous syllable [9]. There exists a feedback pathway from HVC to RA to brainstem vocal and respiratory centers, which then project back to HVC via the thalamic nucleus Uva [26], [52]–[54]. Through this loop, the activation of one syllable chain in HVC may be triggered by the end of the previous syllable, thus linking together HVC chains to generate a sequence of syllables [9] (Figure 9A,B). Because the projections from mesencephalic regions to Uva are bilateral, this loop has been hypothesized to be critical for the bilateral synchronization of the two HVCs [9], [55], [56]. Here we attempt to relate our observations of the effects of HVC cooling to the hypothesis that there are multiple syllable-related chains in HVC.

Perhaps the simplest model one can imagine for how syllable-related chains in HVC might be linked through brainstem/thalamic pathways is that this feedback occupies the entire duration of a gap (Figure 9A). In other words, at the end of each syllable, a signal could be transmitted through the brainstem feedback loop that takes approximately 50–60 ms to return, bilaterally, to activate the next HVC chain immediately before syllable onset. In this ‘gap link’ model, gaps are produced during the period of brainstem feedback that links syllable chains, and the duration of the gaps is controlled by the duration of this feedback. This model suffers from three difficulties.

One possible problem with the ‘gap link’ model is that it suggests that the song motor pathway (HVC and RA) may be silent during gaps. As the model is drawn in Figure 9A, there is a ‘dead time’ between the end of one syllable chain and the activation of the next chain. In contrast to this prediction, it has been shown that HVC and RA both generate sequences of bursting activity during gaps that are largely indistinguishable from those during syllables [8], [57], consistent with the idea that HVC and RA are actively engaged in controlling the duration of gaps. Of course it is possible that the HVC chain continues to be active after syllable offset, even after the initiation of the feedback loop. We will return to this possibility shortly.

A second and more important difficulty with the ‘gap link’ model is the fact that HVC cooling causes gaps to stretch. In the ‘gap-link’ model, the duration of gaps is explicitly controlled by the latency of the brainstem feedback loop, not by circuit dynamics within HVC, thus gaps should not be stretched by HVC cooling. In contrast to this prediction, HVC cooling slows gaps by roughly the same amount as syllables. While in the present experiments we found that gaps were stretched slightly, but significantly, less than syllables (by a factor of 0.81), a previous study [9] found that HVC cooling caused gaps to stretch slightly more than syllables (median gap stretch 1.12 times that of median syllable stretch). The different numerical findings between these two studies may result from the fact that the temperature-dependent stretch of different gaps exhibited much more variability than that of syllables (see also [9]). Nevertheless, together these studies support the conclusion that gaps are stretched by almost the same amount as syllables, in disagreement with the predictions of the ‘gap link’ model.

One important caveat is that this argument depends explicitly on the assumption that the cooling-induced increase in average gap duration is not explained by an increased latency of the brainstem feedback loop with HVC cooling. While we believe this assumption is a reasonable starting point for this discussion, we recognize that it is conceivable that cooling HVC could increase this latency, perhaps by reducing the strength of the hypothesized descending feedback signal. Indeed, slight changes in feedback latency could account for some of the observed variability in gap stretch and the fact that gaps have been observed to stretch slightly more than syllables during HVC cooling (Long and Fee, 2008) and during natural variations in song speed [44].

The finding that HVC and RA exhibit continued activity during gaps, and that HVC cooling causes an increase in gap duration, led Long and Fee (2008) to propose that the duration of gaps is controlled primarily by HVC, perhaps by the same chain-like mechanism hypothesized to control the timing of syllables. Indeed, we can imagine that part of the gap is produced during the feedback loop (e.g., the first part), and the rest of the gap is controlled by the same chain that generates the next syllable. Under the assumption that the latency of the feedback loop is independent of HVC cooling, only the portion of the gap produced by the HVC chain would be slowed by HVC cooling. If we imagine that some fraction of the gap is controlled by HVC, then gaps should exhibit a stretch that is smaller than that exhibited by syllables by this same fraction. The fact that gaps are stretched by almost as much as syllables (∼0.80 in the current study) suggests that at least 80% of the duration of gaps may be controlled by HVC chains. Given that the average duration of gaps is roughly 50–60 ms, the ‘dead time’ between HVC chains might be no more than 10–12 ms, and could be smaller.

Another clue to the nature of the brainstem feedback loop, and one final difficulty with the ‘gap link’ model, comes from electrophysiological recordings. Bilateral multiunit recordings in HVC suggest that the two HVCs receive bilaterally synchronized inputs 43 ms prior to syllable onsets [50] (as well as, occasionally, at other times in the song). These inputs have been hypothesized to synchronize the song motor pathway in the two hemispheres. This is consistent with preliminary findings that HVC-projecting Uva neurons generate a burst of activity approximately 40 ms prior to syllable onsets [58]. While not conclusive, together these findings suggest to us that the HVC chain associated with a particular syllable is activated about 43 ms prior to syllable onset. Thus the activation of the next HVC chain may not occur at the end of the gap, as predicted by the ‘gap link’ model, but nearer to the beginning of the gap.

These arguments suggest that either the latency of brainstem feedback is short, or that the latency is longer but the feedback loop is triggered well before the end of the previous syllable. Interestingly, similar arguments to those made above about the validity of the ‘gap-link’ model can be applied to distinguish these two possibilities. Specifically, the fact that gaps are stretched by almost the same amount as syllables places some constraints, not just on the fraction of the gap controlled by the feedback loop, but also directly on the latency of the feedback loop. These constraints arises if we assume, as before, that the latency of the feedback loop is *not* dependent on HVC temperature, while the portions of the gap controlled by HVC chains *are* dependent on HVC temperature. A mathematical analysis of the effects of HVC cooling on the gap duration shows that the latency (δ) of the feedback loop can be estimated as 
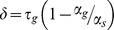
, where. 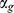 is the temperature dependence of gap stretch, 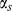 is the temperature dependence of syllable stretch, and 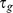 is the gap duration (see Figure S14 for the derivation of this equation). The ratio of gap stretch to syllable stretch we observe here (∼0.8) is consistent with a feedback-loop latency in the range of 10–12 ms. While it is not clear that a latency of 10 ms is physiologically plausible, this calculation suggests that the feedback loop is significantly shorter than the average gap duration. A model with HVC chains connected by a fast brainstem feedback loop is depicted in Figure 9B.

Again, this calculation is based on a number of assumptions, the most important being the temperature independence of the feedback loop latency. Clearly, a more direct experimental test of the latency of the feedback loop would be desirable. One nice experiment, while perhaps not currently feasible, would be to interrupt the descending feedback projections from RA at different times prior to the end of a syllable and determine at which point such interruption reliably inhibits the activation of the next syllable. In summary, we view the arguments above about the nature of HVC chains and brainstem feedback loops not as conclusions or statements of fact, but rather as the framing of a working hypothesis to be vigorously tested. We proceed now to incorporate our observations about respiratory patterns into this framework.

### The relation between respiration and syllable-chains in HVC

As noted above, the timing of bilaterally synchronized bursts in HVC and bursts in Uva suggest that HVC chains may be activated 43 ms prior to the onset of each syllable. This is significantly shorter than the average interval from IP onset to next syllable onset that we observed (average 49.1±3.0 ms), suggesting that the activation of a syllable chain occurs shortly after the onset of the preceding IP (roughly 5–10 ms on average). Thus, it seems likely that the end of one syllable chain in HVC acts to trigger an IP (Figure 9C) and also transmit a signal through the brainstem feedback loop to activate the next syllable chain early in the IP. This argument is based on the *average* latency of the synchronized activity in HVC (or bursting in Uva), and the *average* duration of IPs. There is, of course, variability in the timing of these events for different syllables; a more detailed experimental test of these ideas would require the simultaneous recording of HVC or Uva activity and air sac pressure.

While the relative timing of activity in HVC and Uva suggests to us that IPs are initiated by the end of one HVC chain and terminated by the next HVC chain (Figure 9C), we wish to explicitly compare this view with the alternative possibility that IPs are initiated and terminated by the same HVC chain, either at the beginning of a syllable (Figure 9D), or at the end of a syllable (Figure 9E). We argue that the temporal jitter of different components of the gap support the former view rather than the alternative possibility.

In the model that an IP onset is initiated (‘I’) at the end of the first (‘1’) syllable chain and then terminated (‘T’) ∼40 ms later by the second (‘2’) chain (I1/T2 model, Figure 9C), the duration of the IP itself might be expected to exhibit larger fractional temporal jitter than EPs because, while most of the IP duration is controlled by HVC chains (like EPs), the timing of IP duration also involves a brainstem feedback loop that requires at least four synaptic steps (from HVC back to HVC). The essence of the argument is that this multisynaptic feedback loop is likely to introduce additional jitter into the duration of whichever aspect of song timing it involves. Consistent with the expectation of the I1/T2 model, we found that IP durations have a significantly larger fractional jitter than EPs (5.57% CV of IPs compared to 2.45% CV of EPs). Furthermore, in the I1/T2 model, both the offset of the first syllable and the onset of the subsequent IP are controlled by the first chain, and thus the interval between these two events (the IP onset period) should have small absolute timing jitter. By the same argument, the IP offset period should have a similarly short absolute jitter. As expected in the I1/T2 model, IP onset and offset periods were found to have short absolute jitter (1.32 ms and 1.13 ms, respectively), and these jitters were not significantly different from each other.

In an alternate view, IP onsets and offsets may both be controlled by one syllable chain. For example, one can imagine that at the end of the first syllable chain, HVC terminates the first syllable and simultaneously activates the feedback loop to initiate the second syllable chain (Figure 9D). The next chain then initiates the IP and ∼40 ms later terminates the IP and begins the next syllable. In this model, which we term the ‘I2/T2’ model because the IP is initiated and terminated on the second chain, it is the duration of the EP, not the IP, that depends on propagation through the ‘noisy’ brainstem feedback loop, suggesting that IPs might have less fractional jitter than EPs. Furthermore, in the I2/T2 model, the ‘noisy’ feedback loop is inserted between the end of the first syllable and the IP onset, so the IP onset period might be expected to have significantly more absolute timing jitter than other gap components, particularly the IP offset period. Our observations are not consistent with these expectations. Thus, while arguments based on song variability are not conclusive, our findings are more supportive of the I1/T2 model. Similar logic can be applied to a model in which the IP is initiated and terminated at the end of the first HVC chain (an ‘I1/T1’ model, Figure 9E).

A slightly different version of the I2/T2 model is also worth considering. One can imagine that at the end of the first syllable chain, HVC does *not* terminate the first syllable but simply activates the feedback loop to initiate the second syllable chain. The next chain then terminates the syllable, initiates the IP and ∼40 ms later terminates the IP and begins the next syllable. In this case, the onset and offset of the gap are controlled entirely by the second HVC chain, and should have very low fractional jitter. In contrast, the onset of each syllable is controlled by one chain and the offset is controlled by the next chain, thus introducing additional variability into syllable durations because of the feedback loop. The prediction of this model - that gaps would have a smaller fractional jitter than syllables – is not supported by measurements of gap and syllable jitter [44]. Here again we argue that the existing data are in better agreement with the I1/T2 model.

### HVC control of respiratory gestures during singing: a hypothesis

Our data support a model of respiratory control in which expiratory pressures during singing are controlled continuously by HVC, likely by activation of expiratory brainstem centers such as nucleus retroambigualis (RAm), via descending projections of the motor cortical homologue nucleus RA [14], [46]. Our data also support a model in which IPs are initiated by HVC at the end of each syllable chain and terminated by HVC roughly 40 ms into the following syllable chain. In many cases, the time course of the IP appears to be generated or controlled primarily by circuitry downstream of HVC, perhaps brainstem respiratory centers. In other cases it appears that HVC continues to exert significant control of inspiratory pressure throughout the IP, possibly by direct descending projections of RA to inspiratory centers such as nucleus paraambigualis (PAm) [14], [46] (Figure 9F). It is thought that expiratory and inspiratory centers RAm and PAm likely correspond to the mammalian caudal and rostral ventral respiratory groups (cVRG and rVRG), respectively [45].

We now speculate on some possible brainstem mechanisms that could underlie IP generation, and the means by which HVC and RA could act to initiate and terminate IPs. In addition to projecting to RAm and PAm, RA also projects directly to the ventrolateral nucleus of the rostral medulla (RVL), which may correspond to the mammalian Bötzinger complex [45]. In mammals, the Bötzinger complex (BötC) is thought to contain part of the core microcircuitry that drives the cyclic inspiratory and expiratory pattern of eupneic breathing [59]. This core circuitry has been divided into two functionally distinct and mutually inhibitory components: the BötC circuit that drives transitions to the expiratory part of the cycle, and the pre-BötC circuit that drives transitions to the inspiratory part of the cycle [60].

RA could interact with this respiratory oscillator to initiate IPs in several different ways. First, descending drive to RVL could excite BötC-like neurons, which would act to inhibit the spontaneous generation of inspiratory transitions by pre-BötC-like neurons during the production of a song syllable. Then, at the end of a syllable, HVC and RA could initiate an IP by a sudden reduction in the excitatory expiratory drive to BötC neurons. Recent models of respiratory patterning [60] suggest that this would release pre-BötC neurons from the inhibitory influence of BötC, and result in a fast rebound activation in pre- BötC, thus producing an inspiratory pulse. In this way, descending telencephalic drive could override the natural eupneic oscillatory pattern of the BötC/pre-BötC complex during singing, but still take advantage of the intrinsic inspiratory dynamics of the pre-BötC circuit to drive IPs between syllables. Alternatively, HVC and RA could initiate IPs by directly activating inspiratory bursts in the pre-BötC network by a BötC-independent mechanism, as is thought to occur in gasping [60], [61].

It is also interesting to speculate about the possible involvement of respiratory circuits in the brainstem feedback loop described above. Schmidt and colleagues have recently described a direct projection from PAm to Uva, by which a signal could be transmitted to HVC indicating that an inspiration has been initiated. More specifically, a large burst in the pre-BötC network at IP onset would activate (the rVRG-like) PAm, which could then serve as the source of activity that initiates the next syllable chain in HVC [53].

How does the next HVC chain terminate a brainstem generated IP? The chain could simply time out a 40 ms delay and then strongly activate the descending drive to BötC neurons, which would in turn inhibit the inspiratory pre-BötC network. Together with descending activation of RAm, this would have the effect of driving a rapid transition to positive expiratory pressure and the onset of the next syllable.

Despite the likely involvement of brainstem mechanisms in the generation of inspiratory pulses, it is worth reiterating that some IPs could be directly driven by HVC via the RA to PAm pathway, and bypass altogether the hypothetical brainstem IP generating circuits discussed above. IPs controlled in this way by HVC would be expected to stretch uniformly with HVC cooling. Indeed, such IPs might be produced within a single HVC chain. Such an IP would not be associated with a brainstem feedback loop and could thus exhibit a low duration variability similar to EPs. Localized cooling of the Bötzinger and pre-Bötzinger complex during singing might be used to directly test the involvement of dynamics in brainstem respiratory circuits in the control of IPs.

### Mechanisms of respiratory timing and social context

Song that is directed to a female is performed at a slightly faster tempo (∼3%) than that preformed in an undirected manner [62]–[64]. It has recently been reported that social context has a differential effect on the timing of IPs and EPs [35]. Consistent with the faster tempo, Cooper and Goller (2006) [35] found that EPs recorded during directed song are slightly shorter that those during undirected song. But surprisingly, in contrast to EPs, they found that IPs are not shorter, on average, in directed song than in undirected song. Both of these findings were confirmed in our own data (Figure S10A and B).

In contrast to the differential effects of social context, there appears to be a separate process that has a coordinated effect on the timing of EPs and IPs: EP and IP durations both exhibit a slight increase as the bird repeats multiple renditions of the motif during a bout of singing, an effect that occurs in both directed and undirected song [35], [43]. The surprising differential effect of social context on EP and IP durations, together with the common duration changes during song bouts, led to the suggestion that there are two separate timing mechanisms underlying the control of EP and IP durations [35].

The model presented in Figure 9C already incorporates two separate mechanisms to explain several differential observations about the timing of EPs and IPs. Can this model also be reconciled with the earlier findings of Cooper and Goller? The observation of coordinated slowing of EPs and IPs during song bouts is reminiscent of the coordinated slowing of EPs and IPs with HVC cooling (Figure 2). Both of these observations are consistent with the idea that the timing of respiratory patterns is controlled, at least partly, by circuit dynamics within HVC, and that this mechanism is involved in both social contexts. In this view, the intrabout slowing of EPs and IPs could be explained by a slight ‘fatigue’ during repeated motifs that slows HVC circuit dynamics, much the way HVC is slowed by cooling.

With a few additional assumptions, the model presented in Figure 9C can also explain the effects of social context on song timing. First, changes in song tempo between directed and undirected song are strongly correlated with physiological arousal, such as increased heart rate [35]. In fact, it has recently been observed that the temperature of HVC in a male bird increases by ∼1°C when a female bird is placed nearby (Aronov and Fee, unpublished observations). Note that a 1°C change in HVC temperature would produce the same change in song speed as that typically observed in different social contexts (∼3%). Thus, we hypothesize that one factor in the difference between directed and undirected song is a global arousal mechanism, such as brain temperature, that affects dynamics in HVC and tends to produce a shortening of the duration of both EPs and IPs during directed song.

Of course, the model must still explain the observation that IP durations do not change on average. One possibility is that the midbrain/thalamic link from one syllable to the next is slightly slower in directed song than in undirected song. A slowing of the brainstem feedback loop would tend to increase the duration of IPs in directed song, thus offsetting the effect of physiological arousal (as described above) that would otherwise shorten IPs. In the model shown in Figure 9C, the brainstem feedback loops are not active during EPs, thus, in contrast to IPs, EPs *would* be shortened during directed song. In summary, the additional involvement of a midbrain/thalamic loop in the timing of IPs, but not EPs, provides a possible explanation for the kind of asymmetries observed in the timing of EPs and IPs, and may provide the additional timing mechanism suggested by Cooper and Goller (2006).

### Conclusions

To summarize, we have shown that when executing a learned singing behavior, the zebra finch forebrain is capable of exerting two distinct forms of control over respiratory patterning: 1) precise moment-to-moment control of expiratory pressure during syllables, and 2) the initiation and termination of inspiratory pulses, the dynamics of which appear in many cases to be controlled downstream of HVC, perhaps by brainstem circuits. Our findings suggest a specific model of respiratory-vocal control in adult song, and provide insight into how forebrain motor centers controlling learned behavior exert precise yet flexible control over subcortical motor systems. In addition, our findings provide some insight into how subcortical systems may activate and coordinate subsequent cortical activity through thalamic feedback circuits [65].

## Supporting Information

Figure S1 **Machine drawing of the copper heat sink.** Channels for water flow (highlighted in blue, 0.125 mm width) were cut by a thin saw into a small copper block at 0.4 mm intervals and 0.5 mm depth (0.275 mm fin thickness, 0.5 mm fin height). Small stainless steel tubes (24 gauge, extra-thin walls) were soldered to the block for water inlet and outlet. The slits at the top and bottom are an artifact of the machining process and are sealed with copper foil and solder.
(EPS)

Figure S2 **Song spectrograms and simultaneously recorded thoracic pressure measurements for the five birds used in this study.** Highlighted here with (*) are the syllables with sufficiently complex EP waveforms that were tested for uniformity of stretch via template fitting (Figure 3).
(EPS)

Figure S3 **An example spectrogram and air sac pressure trace for all 22 identified gaps and IPs analyzed in the paper (scale bar = 50 ms).**
(EPS)

Figure S4 **Examples of spectrograms and simultaneously recorded thorasic air sac pressure measurements showing bouts of song surrounded by normal eupneic breathing.**
(EPS)

Figure S5 **HVC cooling slightly reduces EP amplitudes but does not affect IP amplitudes.** A) Average percentage change of the peak pressure (99^th^ percentile) as a function of HVC temperature change for each EP (n = 22 EPs in 5 birds). B) Histogram of the temperature dependence of the peak EP pressure (the slope of the lines plotted in panel A). C) Average percent change of the pressure minimum (1^st^ percentile) of each IP as a function of HVC temperature change (n = 22 IPs in 5 birds). D) Histogram of the temperature dependence of the minimum IP pressure (the slope of the lines plotted in panel C).
(EPS)

Figure S6 **Illustration of how the cumulative stretch of IPs is computed.** A–C) For a given identified IP, the amount of time from IP onset (zero crossing of pressure) to the point at which the IP pressure waveform reached a certain percentage of its minimum (A: 50% descending; B: 90% ascending; C: 40% ascending) was ascertained for every rendition of the IP. This was done separately for each temperature condition (top, middle, and bottom panels). For simplicity, only three renditions are shown in each condition. D–F). For each threshold, the crossing times are normalized by the average time in the control condition. The relationship between these normalized values and the temperature in HVC is assessed by calculating the slope of the line of best fit, which represents the fractional stretch per degree of the portion of the IP prior to the threshold crossing. G) The fractional stretch per degree at each threshold is normalized by the fractional stretch per degree of the entire motif to give the plotted cumulative stretch curve.
(EPS)

Figure S7 **Illustration of how the local stretch of IPs was computed.** Local stretch of IPs was computed in a fashion very similar to how cumulative stretch was computed (see Figure S6). The only difference is that cumulative stretch is determined from the time from the beginning of the IP to a particular percentage of the minimum pressure, whereas the local stretch is determined from the time between two closely spaced (10%) pressure percentages. The remainder of the steps were the same as in Figure S6.
(EPS)

Figure S8 **EP pressure waveform and stretch is preserved following ts-nerve section.** A) Spectrogram and thoracic air sac pressure of a motif from Bird 4 at control HVC temperature prior to ts-nerve transaction. B) Spectrogram and air sac pressure of a motif from Bird 4 at control HVC temperature following ts-nerve transection. The detailed acoustic structure of the song is lost, but the pattern of expiratory pressure is preserved, suggesting that patterned restriction of the airflow by the syrinx does not significantly contribute to the pressure modulations measured in the thoracic air sac. C) The air sac pressure, following ts-nerve transection, with maximal HVC cooling (blue-dashed line) is highly correlated with an artificially stretched version of the control pressure trace (red trace) from part B (r^2^ = 0.94). D–F) Analogous to panels A–C for Bird 7 (panel F: r^2^ = 0.92). G) A comparison of the goodness-of-fit (correlation coefficient) of EP renditions with and without uniform stretching (for the 5 identified EPs considered here. This analysis is analogous to Figure 3B. A stretch-optimized fit of the template to the control and cooled EPs was computed for ts-nerve sectioned birds. This is analogous to the analysis described in the results for non-transected birds. The stretch-optimized fit to the cooled EPs (r^2^ = 0.92±0.06) was nearly as good as the fit to the control EPs (r^2^ = 0.98±0.02), suggesting that linear stretch captures the majority of the waveform changes between EPs in cooled and control conditions – even in ts-nerve transected birds. Furthermore, the ratio of the optimal template stretch to the fractional change in EP duration was not significantly different from one (1.000±0.002, p = 0.87) over the entire range of temperatures (slope as a function of temperature: −0.11±0.088%/°C of cooling, not significantly different from zero, p = 0.28). The shift between the midpoint of the stretched template and the midpoint of the fitted EP waveform, expressed as a percentage of the template duration, was ∼1% over the entire range of temperatures (−1.08±0.27% in the cold condition alone; slope as a function of temperature 0.10±0.04%/°C, p<0.05).
(EPS)

Figure S9 **Respiratory events in directed and undirected song are lengthened equally by HVC temperature change.** A–D) Average duration of both undirected and directed IPs and EPs in Bird 4 at different HVC temperatures, as a percentage of their duration at the control temperature. (A slight horizontal jitter was added to all data points to prevent overlap of error bars.) E–H) Histogram of temperature-dependent stretch of undirected IPs (n = 15), directed IPs (n = 15), undirected EPs (n = 14), and directed EPs (n = 14), relative to the stretch of the song motif of which they were a part (n = 3 birds).
(EPS)

Figure S10 **The effect of temperature on duration is larger than the effect of singing context.** A) The percent change in IP duration during undirected singing relative to directed singing (left column, population not significantly different from 0, p>0.5, t-test). In contrast, cooling HVC produces a large change in IP durations (p<0.001, t-test). B) Analogous to panel A for EPs (p<0.002 and p<0.001 respectively). For both IPs and EPs temperature had a larger effect than singing context (p<0.001 paired t-test in both cases). While IP durations as a population are not consistently longer during undirected singing [35], many individually identified IPs were significantly changed by singing context (blue circles indicate individual EPs and IPs that had duration changes significantly different from zero, p<0.05, t-test). C and E) Examples of two IPs that were signicantly longer during undirected singing. The average waveform of the directed (red) and undirected (black) IPs (top panels). Histogram of durations for directed (red) and undirected (black) IPs (bottom panels, p<0.001 and p<0.003 respectively, t-test). D and F) Examples of two IPs that were significantly shorter during undirected singing (p<0.03 and p<0.01, respectively).
(EPS)

Figure S11 **Local Stretch Analysis of IP waveform changes with HVC cooling.** The analysis here is similar to that shown in Figure 4, except that the local stretch analysis is used rather than cumulative stretch analysis (see Methods and Figure S7). A) Local stretch curves (analogous to Figure 4D) for each IP shown in Figure 4A,B. These IPs exhibited a highly non-uniform stretch, with little temperature dependence during the descending phase of the IP. B) Local stretch curve for all IPs with a uniformity metric less than 0.5 (same IPs shown in Figure 4F). C) Mean and standard deviation of the local stretch curves shown at left (panel B). D) Local stretch curves (analogous to Figure 5C) for each IP shown in Figure 5A,B. E) Local stretch curve for all IPs with a uniformity metric greater than 0.5 (same IPs shown in Figure 5D). F) Mean and standard deviation of the local stretch curves shown at left (panel E). G) Histogram of the center of mass of the local stretch metric for all IPs. Grey shading indicates IPs with a uniformity metric less than 0.5, white indicates those with a uniformity metric greater than 0.5. Values near 100% indicate a center of mass at the center of the IP, as expected for IPs with a perfectly uniform local stretch metric. Values near 50% on the ascending phase indicate that the stretch occurs, on average, halfway through the rising phase of the IP.
(EPS)

Figure S12 **Nonuniform stretch of IPs during natural variations in IP duration.** A) Similar to Figure 4, except only the control temperature condition was included in the analysis, thereby focusing on natural IP duration variations. B–E) Analogous to Figure 4D–G except rather than analyzing cumulative stretch as function of temperature, it is analyzed as a function of total IP duration. In panel C, two IPs have a uniformity greater than 2 (3.15; 3.71) and are not plotted. These outliers are due to a large artifact in the cumulative stretch metric that can occur near the minimum of the IP waveform (see Figure S11C).
(EPS)

Figure S13 **Uniform stretch of IPs during natural variations of IP duration.** A) Analogous to Figure 5A, except only the control temperature condition was included in the analysis, thereby focusing on natural IP duration variation. B–D) Analogous to Figure 5C–E except rather than analyzing cumulative stretch as function of temperature, it is analyzed as a function of total IP duration.
(EPS)

Figure S14
**Schematic diagram of the quantities used to calculate the latency of the brainstem feedback loop.** The latency of the feedback loop is given by δ. The feedback loop is initiated wthin HVC chain 1 at a time 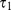 before the offset of the syllable (i.e. gap onset). The feedback loop initiates HVC chain 2 after a latency 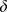. The next syllable begins at a time 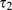. after the onset of HVC chain 2. The total duration of the gap is given by 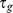. These quantities are thus constrained by the following equation:
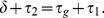
(1)Thus we can write the duration of the gap as
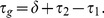
(2)We now assume that the gap duration varies as a function of temperature according to the following relation: 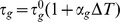, where Δ*T* is the temperature change, 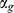 is the coefficient of temperature dependence of gap stretch, and 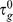 is the gap duration at normal brain temperature. We also assume that the time intervals, 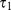. and, 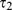 are temperature dependent, because they are controlled by HVC chains 1 and 2, respectively, and that these exhibit the same temperature dependence as song syllables. Thus, we can write 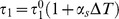., where 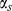 is the coefficient of temperature dependence of syllable stretch, and, 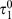. is the duration of the, 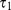 interval at normal brain temperature. A similar relation can be written for, 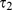. Finally, we assume that the feedback loop latency is independent of HVC temperature.By substitution into Eq. 2, we can now write the equation for the temperature dependent gap duration as:
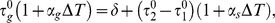
and after expansion we find that
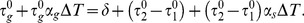
(3)Since we know from Eq. 2 that 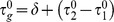.., we can substitute this into the first term on the left side of Eq. 3, and simplify to obtain the following:
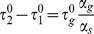
(4)We can now use Eq. 2 to determine that, at normal brain temperature, 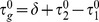. Thus, we can write the feedback loop latency as 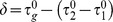. Substitution of Eq. 4 into this equation yields our estimate of the feedback loop latency as a function of the normal gap duration and the ratio of gap to syllable stretch coefficients:
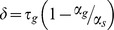

(EPS)
